# *In Vitro* Screening for Anti-Cholinesterase and Antioxidant Activity of Methanolic Extracts of Ayurvedic Medicinal Plants Used for Cognitive Disorders

**DOI:** 10.1371/journal.pone.0086804

**Published:** 2014-01-23

**Authors:** Maya Mathew, Sarada Subramanian

**Affiliations:** Department of Neurochemistry, National Institute of Mental Health and Neurosciences, Bangalore, India; “Mario Negri” Institute for Pharmacological Research, Italy

## Abstract

Inhibition of Acetylcholinesterase (AChE) is still considered as the main therapeutic strategy against Alzheimer’s disease (AD). Many plant derived phytochemicals have shown AChE inhibitory activity in addition to the currently approved drugs for AD. In the present study, methanolic extracts of 20 plants used in Indian Ayurvedic system of medicine for improving cognitive function were screened for acetylcholinesterase inhibitory activity by Ellman’s microplate colorimetric method. Out of 20 extracts, *Emblica officinalis*, *Nardostachys jatamansi*, *Nelumbo nucifera*, *Punica granatum* and *Raulfia Serpentina* showed IC_50_ values <100 µg/ml for acetylcholinesterase inhibitory activity. Antioxidant activities of these plants were assessed by DPPH scavenging assay. Among the extracts used, antioxidant activity was highest for *Terminalia chebula* and *Emblica officinalis* with IC_50_ values <10 µg/ml. Considering the complex multifactorial etiology of AD, these plant extracts will be safer and better candidates for the future disease modifying therapies against this devastating disease.

## Introduction

Discovering improved disease modifying therapies against Alzheimer’s disease (AD) is a major challenge of this century. Neuropathological occurrence of AD symptoms and cognitive deficits is consistent with the presence of cholinergic deficit due to the degeneration or atrophy of cholinergic neurons in the basal forebrain in addition to the senile plaques and neurofibrillary tangles [1]. Thus AChE inhibition has emerged as the major therapeutic target based on this “cholinergic hypothesis”. The only approved drugs for AD- galantamine, rivastigmine and donepezil are acetylcholinesterase inhibitors apart from the NMDA antagonist memantine [2]. Natural products have already proven to be promising sources of useful acetylcholinesterase (AChE) inhibitors [3], [4]. The currently approved drugs for AD, galantamine and rivastigmine are plant derived alkaloids which offer only symptomatic relief without preventing the progression of the disease [5]. AChE inhibition is also considered as a promising therapeutic strategy for other types of dementia, myasthenia gravis, glaucoma and Parkinson’s disease in addition to AD [6]. There is still a need for exploring the nature for newer potent and long lasting AChE inhibitors with minimal side effects. A large number of plant species from different parts of the world have been screened for cholinesterase inhibitory activity [7]–[11].

Another important neurotoxic pathway in AD is based on the formation of reactive oxygen species (ROS), which can cause neuronal injury and death. During aging, oxidative stress is aggravated as a consequence of both an accelerated generation of ROS and a gradual decline in cellular antioxidant defence mechanisms [12] http://www.ncbi.nlm.nih.gov/pmc/articles/PMC3575497/- pone.0056870-Palacios1. Antioxidant therapy has proven to be successful in improving cognitive function and behavioral deficits in patients with mild to moderate AD [13]. Natural products are the biggest source of antioxidants which can be beneficial for AD therapy.

The multifactorial nature of AD suggests that a multi-targeted therapeutic approach might be more advantageous than single- target drugs and combination therapies [14]. Some of the AChE inhibitor molecules currently being evaluated in clinical studies, such as memoquin targets different additional pathways of AD by exhibiting antioxidant functions, acting as β-secretase inhibitor, preventing Aβ aggregation and influencing tau hyperphosphorylation [15]. Natural drug candidates with antiamyloidogenic and antioxidant properties in addition to cholinesterase inhibitory activity could be regarded as especially desirable.

Ayurvedic system of medicine of India has been using various plant species since 4000 years for treating central nervous system disorders as well as to improve the memory and cognitive function [16]–[18]. It will be appropriate to screen these plants in detail in search of new AChE inhibitors as ayurvedic drug combinations from these plants are well tolerated with fewer side effects and possess a definite effect on the CNS. The current study is an attempt to identify and compare the potential candidates from among the plants for cholinesterase inhibition as well as antioxidant activity. 20 different plants were selected which are traditionally used in ayurveda for rejuvenation and for improving the memory and cognitive function (Table 1).

**Table 1:** Details of the Indian plants used in the current study and its traditional uses [16], [17] related to CNS.

Sl. No	Botanical name	Family	Common name	Local name	Traditional uses related to brain [16], [17]
**1**	*Acorus calamus* Linn.	Araceae	Sweet flag	Vacha	against insomnia, insanity, epilepsy, neuropathy
**2**	*Bacopa monniera* (Linn.) Pennell	Scrophulariaceae	Water hyssop	Brahmi	against epilepsy, insanity, memory loss, depression
**3**	*Cedrus deodera* (Roxb.) G.Don	Pinaceae	Deodar cedar	Devadar	against insanity
**4**	*Celastrus paniculatus* Willd.	Celastraceae	Black oil tree	Jyothishmati	to treat mental retardation, epilepsy, anxiety
**5**	*Centella asiatica* Linn.	Apiaceae	Indian pennywort	Gotukola	against insomnia, epilepsy, mental retardation
**6**	*Convolvulus pluricaulis* Choisy.	Convolvulaceae	Bindweed	Shankhapushpi	to enhance memory and intellect
**7**	*Coriandrum sativum* Linn.	Umbelliferae	Coriander	Dhania	to improve vitality and memory
**8**	*Emblica officinalis* Gaertn.	Euphorbiaceae	Gooseberry	Amla	anti-ageing
**9**	*Evolvulus alsinoides* Linn.	Convolvulaceae	Dwarf morning glory	Vishnukranthi	to improve memory
**10**	*Glycyrrhiza glabra* Linn.	Leguminosae	Licorice	Yashtimadhu	to enhance memory
**11**	*Nardostachys jatamansi* DC.	Valerianaceae	Indian spikenard	Jatamansi	against insanity, epilepsy, insomnia, anxiety
**12**	*Nelumbo nucifera* Gaertn.	Nelumbonaceae	Lotus	Kamala	against insomnia, restlessness
**13**	*Punica granatum* Linn.	Punicaceae	Pomegranate	Anar	anti-ageing
**14**	*Rauvolfia serpentina* Linn.	Apocynaceae	Snakeroot	Sarpagandhi	against insanity, epilepsy, insomnia
**15**	*Saussurea lappa* C.B.Clarke.	Asteraceae	Costus	Kushta	against epilepsy, insanity
**16**	*Terminalia chebula* Retz.	Combretaceae	Chebulic myrobalan	Harithaki	to treat neuropathy, general debility
**17**	*Tinospora cordifolia* (Thunb.)Miers	Menispermaceae	Tinospora	Guduchi	anti-ageing
**18**	*Trigonella foenum graceum* Linn.	Fabaceae	Fenugreek	Methi	anti-diabetic
**19**	*Valeriana wallichii* DC.	Valerianaceae	Indian valerian	Tagara	against insomnia, epilepsy, emotional stress
**20**	*Withania somnifera* (Linn.) Dunal.	Solanaceae	Winter cherry	Ashwagandha	rejuvenating nervine tonic

## Materials and Methods

### Chemicals

DPPH, acetylthiocholine iodide (ATCI), AChE from electric eel (type VI-S lyophilized powder), bovine serum albumin (BSA), 5, 5′-dithiobis [2-nitrobenzoic acid] (DTNB), physostigmine, gallic acid and ascorbic acid were purchased from Sigma Aldrich (Bangalore). All other reagents used were of analytical grade and obtained locally.

### Plant Materials

All the plant materials were either procured from Arya vaidya sala, Kottakkal, Kerala or collected from Ernakulam district, Kerala, India and authenticated by Prof. Philomina A Vadakel, Department of Botany, Nirmala College, Muvattupuzha, Kerala, India. No permits were required for these procurements. Botanical names, common name, local name and traditional usages for CNS effect/cognitive functions are summarized in Table 1.

### Preparation of Methanolic Extracts

Freshly collected plant materials were dried in a hot air oven at 55°C and then pulverised. Part of the plant used has been presented in Table 2. To prepare the extract, 5 g each of powdered plant was extracted using soxhlet extraction unit using 70 ml of methanol as the solvent at 60°C and the extract was collected in the upper chamber. Filtered extract was concentrated to evaporate the methanol completely in vacuum centrifuge and the dried extract was stored in −20°C. Yield for each extract was calculated with respect to the starting material (Table 2). Each extract was then solubilised in methanol and 100 mg/ml stock was prepared and used for the assays. The entire study was conducted using single batch of each plant extract to avoid batch-to-batch variation and maximise the product constancy.

**Table 2:** Yield and total polyphenol content of methanolic extract of various plant parts used in the study.

Sl. No.	Botanical name	Parts used	Yield of methanolicextract (mg/g)	Total polyphenol content(mg gallic acid/g)*
**1**	*Acorus calamus*	rhizome	113.3	18±2.4
**2**	*Bacopa monniera*	whole plant	73.2	8.8±0.56
**3**	*Cedrus deodera*	stem bark	64.3	16.1±1.73
**4**	*Celastrus paniculatus*	seeds	122.0	1.2±0.07
**5**	*Centella asiatica*	whole plant	63.5	7.15±0.15
**6**	*Convolvulus pluricaulis*	whole plant	43.9	9.15±1.04
**7**	*Coriandrum sativum*	leaves	10.6	3.27±0.28
**8**	*Emblica officinalis*	whole fruit	76	110±9.4
**9**	*Evolvulus alsinoides*	whole plant	22.4	2.52±0.09
**10**	*Glycyrrhiza glabra*	roots	56.6	9.37±1.2
**11**	*Nardostachys jatamansi*	rhizome	33.8	11.35±1.78
**12**	*Nelumbo nucifera*	flower	70.0	31.5±2.8
**13**	*Punica granatum*	whole fruit	269.7	37±2.9
**14**	*Rauvolfia serpentina*	roots	83.6	14.05±0.98
**15**	*Saussurea lappa*	roots	201.1	5.65±0.07
**16**	*Terminalia chebula*	whole fruit	252.8	93±7
**17**	*Tinospora cordifolia*	stem	20.2	10.85±0.64
**18**	*Trigonella foenum graceum*	seeds	108.0	4.8±0.53
**19**	*Valeriana wallichii*	roots	41.2	11.35±0.93
**20**	*Withania somnifera*	roots	39.4	5.05±0.06

Values are expressed as mean ± SD* (n = 3).

### Estimation of Total Polyphenolic Content

Total polyphenolic content of the plant extracts was estimated using microplate assay method modified from standard procedure using Folin-Ciocalteu reagent [19]. 20 µl each of the 10 mg/ml extracts in methanol and the serial gallic standard solutions in methanol were loaded on a 96 well microplate. Then 100 µl of Folin-Ciocalteu reagent was added to each well, mixed well and waited for 5 min. Again 80 µl of 7.5% sodium carbonate solution was added and mixed well. After covering and keeping it in the dark for 2 hrs, absorbance at 750 nm was measured using Tecan Infinite M 200 microplate reader. Total polyphenols were determined using the standard curve obtained for gallic acid. The estimation of the phenolic compounds in each of the extract was performed in triplicate and the results were expressed as mg of gallic acid equivalents (GAE) per g of dry plant part.

### Determination of Antioxidant Activity by Scavenging Effect on 2, 2′-diphenyl-1-picryl Hydrazyl Radical (DPPH)

Antioxidant potential of the 20 extracts was estimated using modified DPPH free radical scavenging assay in 96 micro-well flat plates [20]. Stock solutions of the extracts were prepared as 1 mg/ml in methanol. Each well was filled in with 200 µl extract in methanol starting from 1000 µg/ml down to the lowest 10 µg/ml. Then, 5 µl of the DPPH solution (2.5 mg/ml in methanol) was added to each well. After keeping the plate in the darkness for 30 minutes, the optical density of each well was read using Tecan Infinite M 200 microplate reader at wavelength 517 nm. Percentage inhibition was calculated using the following formula:
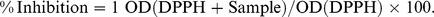



A graph of percentage inhibition of free radical activity was plotted against concentration of crude extract and concentration for 50% inhibition (IC_50_) was obtained from the graph. The radical scavenging effect was examined and compared with other natural antioxidants gallic acid and ascorbic acid which were used as positive controls.

### Determination of Acetylcholinesterase Inhibitory Activity using Microplate Assay

AChE activity was measured using a modified 96-well microplate assay [11] based on Ellman’s method [21]. The enzyme hydrolyses the substrate acetylthiocholine resulting in the product thiocholine which reacts with Ellman’s reagent (DTNB) to produce 2-nitrobenzoate-5-mercaptothiocholine and 5-thio-2-nitrobenzoate which can be detected at 412 nm. 50 mM Tris–HCl pH 8.0 was used as a buffer throughout the experiment unless otherwise stated. AChE used in the assay was from electric eel (type VI-S lyophilized powder, 518 U/mg solid, 844 U/mg protein). The enzyme stock solution (518 U/ml) was kept at −80°C. The further enzyme-dilution was done in 0.1% BSA in buffer. DTNB was dissolved in the buffer containing 0.1 M NaCl and 0.02 M MgCl_2_. ATCI was dissolved in deionized water. In the 96-well plates, 100 µl of 3 mM DTNB, 20 µl of 0.26 U/ml of AChE, and 40 µl of buffer (50 mM tris pH 8.0), 20 µl of each extract in various concentrations (25, 50, 100, 250 and 500 µg/ml) dissolved in buffer containing not more than 10% methanol were added to the wells. After mixing, the plate was incubated for 15 min (25°C) and then the absorbance was measured at 412 nm in Tecan infinite 200 microplate reader and the readings were used as blank. The enzymatic reaction was initiated by the addition of 20 µl of 15 mM ATCI and the hydrolysis of acetylthiocholine was monitored by reading the absorbance every 5 min for 20 min. Physostigmine was used as positive control. All the reactions were performed in triplicate. The percentage inhibition was calculated as follows:
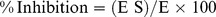



Where; E is the activity of the enzyme without extract and S is the activity of enzyme with the extract. IC_50_ value could be calculated from the % inhibition values of different concentrations of each plant extract.

### Statistical Analysis

Data were expressed as mean ± standard deviation for separate groups for determinations in triplicates. The correlation between the values of the total polyphenolic content of the extracts and their antioxidant activity were analyzed using the Pearson test. IC_50_ values were calculated via non-linear regression analysis (sigmoidal fitting with variable slope) using GraphPad Prism v. 5.0 (GraphPad software Inc., USA).

## Results and Discussion

Acetylcholinesterase (AChE) is a key enzyme in the cholinergic nervous system. Therapies designed to reverse the cholinergic deficit in AD is mostly based on inhibitors of AChE, which enhance cholinergic transmission with modest and transient therapeutic effects. Several studies revealed that cholinesterase inhibitors could act on multiple therapeutic targets such as prevention of the formation of β-amyloid plaques, antioxidant activity and modulation of APP processing [14]. However, there is still a need for new AChE inhibitor lead compounds with lower toxicity and higher central nervous system (CNS) penetration. Many plants have been studied by bioassay-guided approaches for the identification of new AChE inhibitors [3]–[11] and different classes of plant-derived natural products have been considered as new AChE inhibitors potentially useful for AD treatment.

Ayurveda, the ancient Indian system of medicine, dates back to 2000 BC in which various plants effective for treating CNS disorders and aging are well documented [16]–[18]. In the present study, methanolic extracts of 20 such plant materials considered to be ‘nootropic’ or brain boosting were prepared and evaluated for their anti-cholinesterase and anti-oxidant effects.

### Total Polyphenol Content of the Methanolic Extracts

Phenolic compounds have been shown to be responsible for the antioxidant activity of plant materials [22] and hence many of the natural polyphenols possess therapeutic potential for AD. Therefore, the amount of total phenols in the methanolic plant extracts was investigated by the Folin-Ciocalteu method which is expressed as gallic acid equivalents (GAE/g extract) in Table 2. Highest polyphenol content was observed for *Emblica officinalis* and *Terminalia chebula,* with values 110±9.4 and 37±2.9 mg GAE/g of the extract respectively. These two plants are constituents of ayurvedic formulation Triphala along with *Terminalia bellerica* [23]. Higher levels of polyphenolic content were also observed for *Punica granatum* and *Nelumbo nucifera* (pink flower) with corresponding values of 37±2.9 and 31.5±2.8 mg GAE/g.

### Free Radical Scavenging Activity of the Extracts using the DPPH Assay

The antioxidant activity of various methanolic plant extracts were determined using the free radical 1,1-diphenyl-2- picrylhydrazyl (DPPH) by the addition of various concentrations of extracts to DPPH. The amount of remaining DPPH was determined at 30 min based on the absorbance at 517 nm. % Inhibition of DPPH scavenging was calculated for each concentration of individual plant extracts. IC_50_ values for DPPH radical scavenging was determined as the concentration of the extract required to bring about 50% of the original activity. Both the % inhibition of free radical scavenging activity at 100 µg/ml and IC_50_ for all the 20 different methanolic plant extracts are presented in Table 3. The antioxidant activities of the methanolic extracts were correlated to the total polyphenolic content using Pearson’s test (r = 0.7456, P<0.05). The lowest IC_50_ values were obtained for *Emblica officinalis* (7±0.9 µg/ml) and *Terminalia chebula* (8.5±0.5 µg/ml) whereas the values for the positive controls ascorbic acid and gallic acid were 2.5±0.18 µg/ml and 1±0.13 µg/ml respectively. *Emblica officinalis* and *Terminalia chebula* had the highest polyphenol content in the Folin’s assay. These fruits contain ascorbic acid and polyphenols like gallic acid and ellagic acid which are responsible for its antioxidant activity [23]. Methanolic extracts of *Nelumbo nucifera*, *Punica granatum* and *Rauvolfia serpentina* also showed high antioxidant activity with IC_50_ values 45±3.5, 54.5±7.2 and 96±7.8 µg/ml respectively. *Bacopa monniera, Cedrus deodera* and *Glycyrrhiza glabra* also exhibited very good activity for DPPH scavenging with corresponding IC_50_ values of 115±27.8, 116±17.2 and 145±12.3 µg/ml.

**Table 3:** % Inhibition and IC_50_ values of methanolic plant extracts for DPPH scavenging and AChE inhibition assays.

Sl. No.	Botanical name	% Inhibition of DPPHat 0.1 mg/ml*	IC_50_ for DPPH scavengingassay (µg/ml)*	IC_50_ for AChE inhibitionassay (µg/ml)*	% AChE inhibitonat 0.1 mg/ml*
**1**	*Acorus calamus*	10.6±2.1	615±41.2	176±9.6	43.79±7.2
**2**	*Bacopa monniera*	47.5±5.9	115±27.8	523±39.7	15.15±0.97
**3**	*Cedrus deodera*	47.4±6.4	116±17.2	>1000	7.13±0.89
**4**	*Celastrus paniculatus*	3.4±0.4	943±51.2	>1000	23.13±2.3
**5**	*Centella asiatica*	24.5±5.1	270±18.9	890±67.4	30.7±2.9
**6**	*Convolvulus pluricaulis*	21.9±3.2	275±22.1	234±38	40.6±5.4
**7**	*Coriandrum sativum*	0.21±0.1	>1000	>1000	36.25±5.3
**8**	*Emblica officinalis*	86±9.2	7±0.9	53.5±8.9	73.3±5.9
**9**	*Evolvulus alsinoides*	23.6±5.3	280±16	245±32.4	38.03±3.5
**10**	*Glycyrrhiza glabra*	33.5±4.2	145±12.3	418±30.7	35.01±4.6
**11**	*Nardostachys jatamansi*	5.5±1.9	295±15.7	40.5±7.1	83.83±9.2
**12**	*Nelumbo nucifera*	84±10.2	45±3.5	76±9.2	61.73±7.6
**13**	*Punica granatum*	86±9.6	54.5±7.2	77±6.2	62.4±5.3
**14**	*Rauvolfia serpentina*	54.2±4.3	96±7.8	22±4.9	84.9±9.5
**15**	*Saussurea lappa*	5.32±0.3	510±32	>1000	12.15±9.7
**16**	*Terminalia chebula*	86.3±11.4	8.5±0.5	180±14.6	41.06±5.6
**17**	*Tinospora cordifolia*	2.7±0.4	410±36.6	230±17.95	27.75±3.5
**18**	*Trigonella foenum graceum*	5.42±0.5	830±91	>1000	6±0.9
**19**	*Valeriana wallichii*	23.8±3.2	215±13.4	715±63.7	8.82±0.7
**20**	*Withania somnifera*	7.6±1.2	850±65	124±9.8	44.8±4.3

Values are expressed as mean ± SD* (n = 3). IC_50_ for DPPH scavenging assay of positive controls gallic acid and ascorbic acid were 1±0.13 µg/ml and 2.5±0.18 µg/ml respectively. IC_50_ for physostigmine control for AChE inhibition assay was 0.075±0.003 µg/ml.

### Acetylcholinesterase Inhibitory Activity of the Extracts

**Figure 1:**
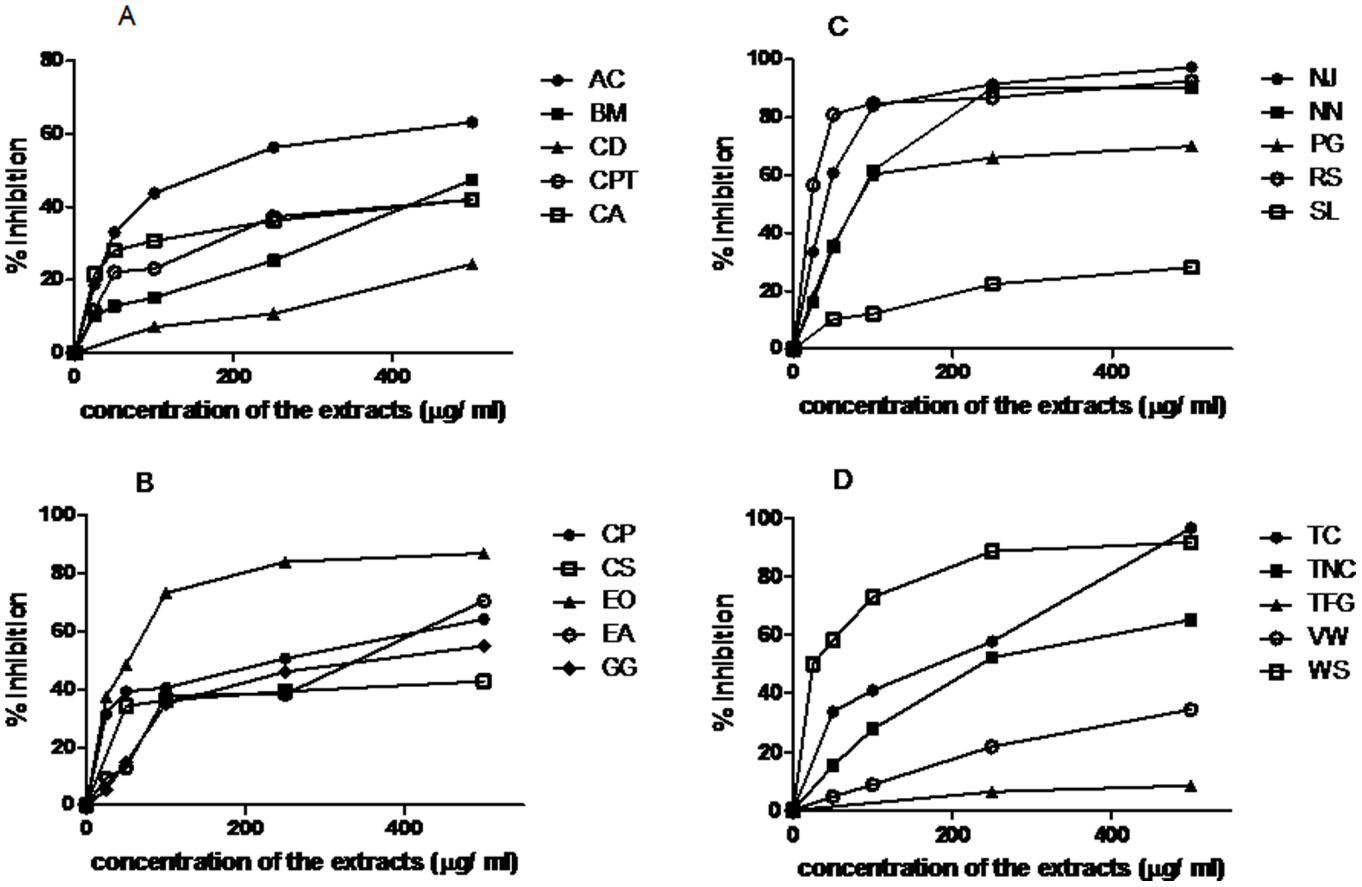
Inhibition of AChE activity by plant extracts. Panel (A) shows the inhibition curves obtained with *Acorus calamus* (AC), *Bacopa monniera* (BM), *Cedrus deodara* (CD), *Celastrus paniculatus* (CPT) and *Centella asiatica* (CA). Panel (B) includes the results with *Convolvulus pluricaulis* (CP), *Coriandrum sativum* (CS), *Emblica officinalis* (EO), *Evolvulus alsinoides* (EA) and *Glycyrrhiza gabra* (GG). Panel (C) represents the inhibition observed with Nardostachys jatamansi (NJ), *Nelumbo nucifera* (NN), *Punica granatum* (PG), *Rauvolfia serpentine* (RS) and *Saussurea lappa* (SL). Panel (D) shows the inhibition curves obtained with the methanolic extracts of *Terminalia chebula* (TC), *Tinospora cordifolia* (TNC), *Trigonella foenum graceum* (TFG), *Valeriana wallichii* (VW) and *Withania somnifera* (WS). Mean values of 3 independent experiments have been plotted.

The methanolic extracts of 20 plants used in Ayurveda for nervous system disorders were tested for AChE inhibitory activity using Ellman’s colorimetric method in 96-welled microplate. The inhibition curves have been presented in Figure 1 (A–D). The results are shown in Table 3 representing the % inhibition at 100 µg/ml and IC_50_ for different extracts. Physostigmine was used as the standard AChE inhibitor in this study which showed IC_50_ of 0.075±0.003 µg/ml. IC_50_ for AChE inhibitory activity was lowest for *Rauvolfia serpentina* followed by *Nardostachys jatamansi, Emblica officinalis, Nelumbo nucifera and Punica granatum* with values 22±4.9, 40.5±7.1, 53.5±8.9, 76±9.2 and 77±6.2 µg/ml respectively. These plants showed very high antioxidant activity also in the DPPH assay. Considerably lower IC_50_ values were exhibited by *Withania somnifera* (124±9.8 µg/ml), *Acorus calamus* (176±9.6 µg/ml) and *Terminalia chebula* (180±14.6 µg/ml).

### Plant Derived Multitargeted Drug Approach for AD Therapy

Out of the 20 plant extracts screened, extracts of dry plant parts from *Emblica officinalis* (fruit), *Nardostachys jatamansi* (rhizome), *Nelumbo nucifera* (flower), *Punica granatum* (fruit), *Rauvolfia serpentina* (root) and *Terminalia chebula* (fruit) were selected as efficacious candidates as sources of potent AChE inhibitors as well as antioxidants. Due to the multifactorial pathogenesis of the AD, multitargeted drugs will be preferred as the effective therapeutic strategy against AD. These plant species used traditionally for treating CNS disorders exhibited *in vitro* AChE inhibition and antioxidant activity further validating their use in traditional medicine. Those that did not inhibit AChE may be acting on a different molecular target related to their traditional use [24]. Among these, *Nardostachys jatamansi* and *Emblica officinalis* were effective in dissociation of the preformed Aβ oligomers whereas the former along with *Terminalia chebula and Emblica officinalis* were effective in inhibition of oligomerisation *in vitro* from our previous investigations [25].

*Emblica officinalis* or Indian gooseberry is used in ayurveda both as a medicine and as a rejuvenating tonic to build up lost vitality and vigour. The methanolic extract from the dried fruits of this plant had highest amount of total polyphenols and antioxidant activity among the 20 plants screened. According to the Ayurvedic text books, Charaka Samhita and Sushruta Samhita, it is regarded as the best among rejuvenative herbs. *Emblica officinalis* is a major ingredient in several important medicinal preparations including Triphala (“three fruits”) which possess anti-aging properties and the Chyawanprash, a general tonic for people of all ages, which improves mental and physical well being. Memory enhancing property was observed for Anwala churna, an ayurvedic preparation based on *Emblica officinalis* in a study on young and aged rats [26]. Methanolic extract of dried fruits of *Emblica officinalis* exhibited IC_50_ value of 53.5±8.9 µg/ml for AChE inhibition which might be owing to its high gallic acid and ellagic acid content [27].

Fruits of *Terminalia chebula* is also used along with *Emblica officinalis* and *Terminalia bellerica* in the ayurvedic formulation called Triphala as a general tonic for well being. Methanolic extract of this fruit also possesses high antioxidant activity due to the presence of gallic acid and ellagic acid [23]. This extract showed moderate levels of cholinesterase inhibition also with IC_50_ of 180±14.6 µg/ml which is relevant for its potential for treating AD.

Roots and rhizomes of *Nardostachys jatamansi* have medicinal value which contains a variety of sesquiterpenes and coumarins. It is mainly used in ayurveda to treat insanity, insomnia, epilepsy, and anxiety. An alcoholic extract of this plant administered to both young and aged mice significantly improved learning and memory and also reversed aging induced amnesia due to diazepam and scopolamine [28]. *Nardostachys jatamansi* extract prevented chronic restraint stress-induced learning and memory deficits in a radial arm maze task [29]. In another study, hydroalcohol extract of *Nardostachys jatamansi* rhizome showed AChE inhibitory activity with an IC_50_ value of 130.11±12 µg/ml [7]. In our observations, methanolic extract of the plant showed an IC_50_ value of 40.5±7.1 µg/ml with moderate antioxidant activity.

*Nelumbo nucifera* or ‘sacred lotus’ is believed to possess CNS enhancing properties and the flowers are used in many of the Ayurvedic formulations. Dried whole pink flowers of lotus was used in our study to make the methanolic extract which gave an IC_50_ value of 76±9.2 µg/ml for AChE inhibition. It had higher total polyphenol content and high antioxidant activity due to the presence of polyphenolics like catechins and procyanidins which also have high therapeutic potential [30]. It is a well studied plant for its anticholinesterase activity [7], [11], [31], [32]. In a previous study a hydroalcohol extract of *Nelumbo nucifera* rhizome showed weak AChE inhibitory activity with an IC_50_ value of 185.55±21.24 µg/ml [7]. Neferine and nuciferine are important alkaloids from the seeds of lotus which showed antiamnesic, antidepressant and sedative activity [30], [31].

Methanolic extracts of dry fruits of *Punica granatum* showed IC_50_ value of 77±6.2 µg/ml for AChE inhibition showing the efficacy of this fruit in enhancing cognitive skills. Pomegranate is well known for its antioxidant activity and anti aging potential due to the presence of punicalgins [33]. Pomegranate juice decreased amyloid load and improved behavior in a APPsw/Tg2576 transgenic mouse model of Alzheimer’s disease [34]. Ellagic acid and punicalgin from this fruit were found to be potent β-secretace inhibitors [35].

Roots of *Rauvolfia serpentina* is mainly used in ayurveda for the treatment of hypertension and psychotic disorders like schizophrenia, anxiety, insomnia, insanity and snakebite. Various indole alkaloids have been identified in these roots and reserpine is the most well known one [36]. Reserpine was found to be ameliorating Aβ toxicity in the Alzheimer’s disease model in *Caenorhabditis elegans* [37]. Methanolic extract of *Rauvolfia serpentina* showed the highest inhibition against AChE with an IC_50_ value of 22±4.9 µg/ml. It also exhibited good antioxidant activity validating the traditional use of this plant.

## Conclusions

The methanolic extracts of plants used in traditional ayurvedic system of medicine of India for improving the memory and cognitive function were screened for AChE inhibition and antioxidant activity. Of the 20 plant materials tested, the methanolic extracts from fruits of *Emblica officinalis,* rhizome *of Nardostachys jatamansi*, flower of *Nelumbo nucifera*, fruit of *Punica granatum,* root of *Rauvolfia serpentina* and fruit of *Terminalia chebula* were selected as promising candidates as sources of potent AChE inhibitors as well as antioxidants. Among these, fruits of *Emblica officinalis* and *Punica granatum* can be consumed daily in our diet for its neuroprotective effect. Since these extracts are able to act on multiple therapeutic targets of AD, further evaluation is required to identify the active ingredients, assess their safety and bioavailability in *in vivo* animal models.
